# Solid−gas carbonate formation during dust events on Mars

**DOI:** 10.1093/nsr/nwac293

**Published:** 2023-01-11

**Authors:** Wenshuo Mao, Xiaohui Fu, Zhongchen Wu, Jiang Zhang, Zongcheng Ling, Yang Liu, Yu-Yan Sara Zhao, Hitesh G Changela, Yuheng Ni, Fabao Yan, Yongliao Zou

**Affiliations:** Shandong Key Laboratory of Optical Astronomy and Solar-Terrestrial Environment, School of Space Science and Physics, Institute of Space Sciences, Shandong University, China; Shandong Key Laboratory of Optical Astronomy and Solar-Terrestrial Environment, School of Space Science and Physics, Institute of Space Sciences, Shandong University, China; CAS Center for Excellence in Comparative Planetology, China; Shandong Key Laboratory of Optical Astronomy and Solar-Terrestrial Environment, School of Space Science and Physics, Institute of Space Sciences, Shandong University, China; Shandong Key Laboratory of Optical Astronomy and Solar-Terrestrial Environment, School of Space Science and Physics, Institute of Space Sciences, Shandong University, China; Shandong Key Laboratory of Optical Astronomy and Solar-Terrestrial Environment, School of Space Science and Physics, Institute of Space Sciences, Shandong University, China; CAS Center for Excellence in Comparative Planetology, China; State Key Laboratory of Space Weather, National Space Science Center, Chinese Academy of Sciences, China; International Center for Planetary Science, College of Earth Science, Chengdu University of Technology, China; CAS Center for Excellence in Comparative Planetology, China; J’Heyrovski Institute of Physical Chemistry, Czech Academy of Sciences, Czech Republic; Department of Earth & Planetary Science, University of New Mexico, USA; Shandong Key Laboratory of Optical Astronomy and Solar-Terrestrial Environment, School of Space Science and Physics, Institute of Space Sciences, Shandong University, China; School of Mechanical, Electrical & Information Engineering, Shandong University, China; State Key Laboratory of Space Weather, National Space Science Center, Chinese Academy of Sciences, China

## Abstract

Electrostatic discharge experiments under simulated martian atmospheric conditions indicate that atmospheric CO_2_ has been sequestered into carbonate by the Mars dust activities during the Amazonia era.

For half a century, Mars missions have gathered abundant geological evidence for a wet, warm climate in the early history of Mars more than 3 billion years ago, including various fluvial landforms and exposed aqueous alteration minerals. It was supposed that Mars once had a dense, CO_2_-rich atmosphere. However, present-day Mars has a much thinner atmosphere dominated by CO_2_. Carbonates were long ago proposed as solid sinks for the dense CO_2_ atmosphere early in the history of Mars. To date, carbonate minerals have been identified in various locations on Mars by remote sensing and rovers and from martian meteorites. Carbonate-bearing rocks on Mars have been found in several small, localized outcrops, which mostly tie towards subsurface hydrothermal settings. It has been widely accepted that these carbonates are products of aqueous processes on Mars, although their specific formation conditions (a short- or long-lived aqueous system, temperature, water:rock ratio) require further constraints.

Trace amounts of carbonate have been found in modern global dust by both orbiters [1] and rovers [2] on Mars, yet their sources and formation mechanisms are unclear. Earth-based thermal emission observations of Mars gave the first indications of carbonates in martian dust. The thermal emission spectrometer aboard the Mars Global Surveyor (MGS) identified minor Mg-rich carbonates (∼2–5 wt.%) in global martian dust [1]. Carbonate has also been found to be a trace to minor component (up to 6 wt.%) in martian soil or regolith around the landing sites of the *Phoenix* Lander, *Spirit* rover, and *Curiosity* rover [2]. Apparently, these carbonate minerals in martian soil/dust can be attributed to detrital sediments eroded by aeolian/fluvial processes or impact events targeted on carbonate-bearing bedrocks. However, carbonate also occurs in present-day Mars global dust, while ancient massive carbonate outcrops are rare on Mars, raising uncertainty on the sources of carbonates in martian dust. Martian atmospheric CO_2_ appears to have been fixed in carbonate dust, which may have played a role in its evolution from a denser to thinner atmosphere [3].

Mars dust events (dust storms, dust devils, and grain saltation) are active on present-day Mars, which could introduce complex chemical reactions by taking surface materials into the atmosphere [4]. In our previous experiments, carbonate and perchlorate were both formed in simulated electrostatic discharge (ESD) reactions with halite [4], but the mineralogy and formation mechanism of carbonates are not well understood.

Here, we used a Mars chamber to simulate an ESD occurring during martian dust activity. Pure CO_2_ with an atmospheric pressure of 3 mbar as well as the Mars Simulate Gas Mixture (MSGM, composed of 95.94% CO_2_, 1.96% Ar, 1.94% N_2_, and 0.16% O_2_) was filled into the chamber to provide a Mars-like atmosphere. More details on the experimental setups and parameters can be found in the Supplementary Materials. Common minerals in the martian atmospheric dust were chosen as the starting materials, such as silicates (olivine, plagioclase, pyroxene, and saponite), Ca sulfates (gypsum), halogen minerals (NaCl, MgCl_2_, NaBr) and (per)chlorate (NaClO_3_, NaClO_4_). These minerals were exposed to ESDs for various durations (30 min, 1 hour, 3 hours, and 5 hours).

Carbonates as well as other new phases were identified using Raman spectroscopy and mid-IR attenuated total reflectance (ATR) spectra after the ESD reactions with halogen minerals and (per)chlorate (Table 1). A sharp and intense peak at 1080 cm^−1^ appeared in the Raman spectrum of the ESD products (Figs S2a, S2b and S3c), which was assigned to the ν_1_ symmetric stretching mode of CO_3_^2−^ in carbonates. In the ATR spectra of the final ESD products, a strong feature at ∼1430 cm^−1^ and a weaker band at ∼883 cm^−1^ (Figs S3 and S4) were assigned to the ν_3_ and ν_2_ fundamental vibrational modes of CO_3_^2−^, respectively. Furthermore, CO_3_^2−^ was not detected in experiments using silicates and Ca sulfates as starting materials (Fig. S5). We also quantified the yields of carbonates formed in the ESD process with a traditional double indicator method (Table S1).

By combining this study and previous experiments [4,5], we attempted to understand carbonate formation during the ESD process. The entire chemical pathway for carbonate formation can be divided into four steps (Fig. 1). First, when energetic electrons produced by the ESD collided with halogen minerals (NaCl and NaBr) and (per)chlorate (NaClO_3_, NaClO_4_) targets, gaseous species (Br^0^, Cl^0^, Cl^+^, Cl_2_^+^, etc.) were likely released from the halogen crystals due to decomposition of the V_K_ centres and sputtering of Na^0^, Na^+^, etc. We speculate that gaseous species and different cations (K^+^, Ca^2+^ Mg^2+^, etc.) were likely released when MgCl_2_, CaCl_2_, FeCl_3_, KCl, KBr and other halogen minerals were used as starting materials. Second, the ESD process further led to ionization or dissociation of gases (CO_2_, N_2_, Ar, etc.), which generated large amounts of free radicals (CO_2_^+^, CO^+^, O_I_, H_III_, H_II_, OH, Ar_I_, N_2_^+^, etc.). Third, the activated free radicals (Cl·, Br·, CO_2_^+^, CO^+^) could be further oxidized by oxidants (e.g. O_3_ and O^2−^ radicals), and carbonate would be produced simultaneously. Finally, CO_3_^2^^−^ ions could combine with metal ions (Na^0^, Na^+^, K^+^, Ca^2+^ Mg^2+^, etc.) to form carbonate deposits on the surface of the material.

**Figure 1.**
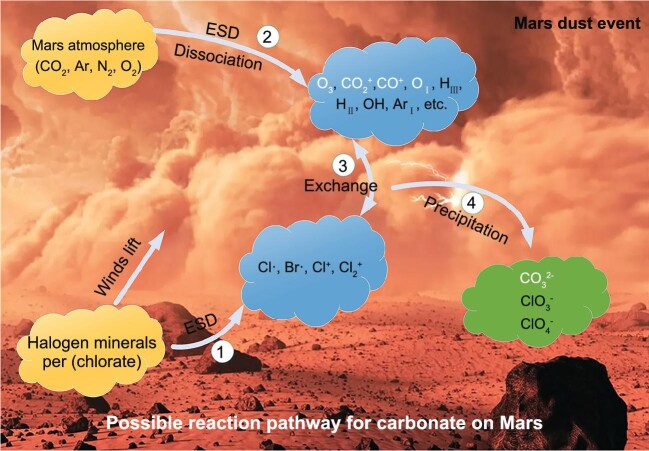
Proposed reaction pathway for carbonate formation by ESD reactions. The pathway for formation of carbonates includes four steps: (1) gaseous species are released from halogens and/or per(chlorate); (2) atmospheric ionization or dissociation occurs; (3) a gas phase reaction occurs between free species (CO_2_^+^, CO^+^) and different oxidants to produce different carbonate species; and (4) carbonates are deposited on the surface materials of Mars. The bottom image of a martian dust storm comes from the Getty Images/Science Photo Library.

**Table 1.** Identification of carbonates in the top layer of each ESD product.

	Raman	Mid-Infrared
Sample	spectroscopy	spectroscopy
NaCl-ESD products	√	√
NaBr-ESD products	√	√
NaClO_3_-ESD products	√	√
NaClO_4_-ESD products	×	√
MgCl_2_-ESD products	—	√
plagioclase-ESD products	—	×
pyroxene-ESD products	—	×
saponite-ESD products	—	×
gypsum-ESD products	—	×
olivine-ESD products	—	×

Notes: ‘√’ means ‘carbonate confirmed’. ‘×’ indicates ‘not detected’. ‘–’ refers to ‘not analysed in the present study’.

Various explanations have been proposed for carbonate formation on Mars, such as chemical deposition in a warm/wet climate on early Mars, authigenic precipitation in a lake and alteration in a subsurface hydrothermal system caused by magmatic or impact activity. Several possible active processes involving atmospheric CO_2_ could also result in carbonate formation on present-day Mars, such as carbonate growth on silicate particles under simulated martian conditions (CO_2_ atmosphere) [6], CO_2_ interactions with liquid water films on dust particles [7], or heterogeneous atmospheric chemical reactions on aerosol surfaces [8]. In all of the mechanisms proposed above, liquid water is clearly the essential factor for carbonate formation. However, our experiments demonstrate solid−gas carbonate formation by ESD on martian dust, perhaps occurring on present-day Mars.

Our study implies that carbonates could be constantly sinking on the surface of Mars in trace quantities from its CO_2_-rich atmosphere, regardless of the water content and relative humidity conditions. Under such circumstances, atmospheric CO_2_ appears to have been sequestered as carbonates across long geologic periods. Carbonate phases would accumulate within martian soils/dusts due to the repeated annual dust events on Mars and then be distributed by martian global atmospheric circulation and martian global dust storms. For example, the presence of carbonate in martian dust, which was detected by orbital thermal emission spectroscopy [1], could be a result of this reaction. Both carbonate and (per)chlorate were detected in the same aeolian deposit samples (Rocknest and Gobabeb) [2], which is consistent with our proposed ESD formation hypothesis (Figs S2 and S3). This observation further argues that the ESD mechanism may account for important processes in carbonate formation on Mars.

Our analyses showed that chloride (as well as bromine), a typical evaporite mineral on Mars as well as a significant component of martian soils/dusts, may serve as a precursor for carbonate formation in the ESD reaction. The gamma ray spectrometer (GRS) aboard Mars Odyssey revealed a heterogeneous Cl distribution on the surface of Mars and obtained a global mean value of 0.49 wt% Cl [9]. Mars rovers made *in situ* observations at different locations (Gale crater, Gusev crater and Meridiani Planum), which indicated that bromine abundances vary by several orders of magnitude and, in turn, dominate the large variations in Cl/Br ratios [10]. Furthermore, (per)chlorate was detected at three distant locations between the *Phoenix, Curiosity* and *Perseverance* landing sites. Based on ESD experiments with different minerals and previous studies [3,4], we demonstrate that electrochemical processes, occurring during simulated Mars dust events, provided a novel pathway to carbonate formation, and halide salts (including Cl^−^ and Br-bearing NaCl, KCl, MgCl_2_, NaBr, KBr, etc.) and (per)chlorate (NaClO_3_, NaClO_4_) acted as precursors for carbonate formation not involving aqueous processes. Atmospheric CO_2_ on Mars appears to have been solidified as carbonates from at least the early Amazonian period to the present day. The total amount of CO_2_ sequestered by Mars dust storms in the Amazonia era has been estimated based on the carbonate yield of NaCl-ESD 5 hour experiments. In the past 3.0 Ga, Mars dust storms possibly converted ∼0.56 mbar of CO_2_ into carbonate (more details can be found in the Supplementary Materials).

## Supplementary Material

nwac293_Supplemental_File

## References

[1] Bandfield JL , Glotch TD, Christensen PR. Science 2003; 301: 1084–7. 10.1126/science.1088054 12934004

[2] Sutter B , McAdam AC, Mahaffy PR et al. J Geophys Res Planets 2017; 122: 2574–609. 10.1002/2016JE005225

[3] Jakosky BM . Planet Space Sci 2019; 175: 52–9. 10.1016/j.pss.2019.06.002

[4] Wu Z , Wang A, Farrell WM et al. Earth Planet Sci Lett 2018; 504: 94–105. 10.1016/j.epsl.2018.08.040

[5] Mao WS , Fu XH, Wu ZC et al. Earth Planet Sci Lett 2022; 578: 117302. 10.1016/j.epsl.2021.117302

[6] Stephens SK . Carbonate Formation on Mars: Experiments and Models. Pasadena: California Institute of Technology, 1995.

[7] Boynton WV , Ming DW, Kounaves SP et al. Science 2009; 325: 61–4. 10.1126/science.1172768 19574384

[8] Shaheen R , Abramian A, Horn J et al. Proc Natl Acad Sci USA 2010; 107: 20213–8. 10.1073/pnas.1014399107 21059939 PMC2996665

[9] Keller JM , Boynton WV, Karunatillake S et al. J Geophys Res-Planets 2006; 111: E03S08. 10.1029/2006JE002679

[10] Wang XY , Zhao YYS, Hood DR et al. J Geophys Res Planets 2021; 126: e2021JE006929. 10.1029/2021JE006929

