# Supplementary material for: Solid−gas carbonate formation during dust events on Mars
Source: Natl Sci Rev. 2023 Jan 11;10(4):nwac293. doi: 10.1093/nsr/nwac293 (PMC10029838; doi:10.1093/nsr/nwac293)
Supplement: nwac293_Supplemental_File [file nwac293_supplemental_file.docx]

**Supplementary Materials for**

**“Solid−gas carbonate formation during dust events on Mars”**

**Wenshuo Mao^1^, Xiaohui Fu^1,4^*, Zhongchen Wu^1^*, Jiang Zhang^1^, Zongcheng Ling^1,4^, Yang Liu^2^, Yu-Yan Sara Zhao^3,4^, Hitesh G. Changela^5,6^, Yuheng Ni^1^, Fabao Yan^7^, Yongliao Zou^2^**

This Word file includes four sections:

1) Details of experimental methods;

2) Possible effects of the atmosphere and air pressure on the ESD reaction;

3) Estimated amounts of CO_2_ sequestered by the EDS process;

4) Figures, tables and their captions.

**1. Detailed experimental methods**

The ESD experiments in this study were focused on new phases produced from halogen minerals and (per)chlorates at different times. Silicates (olivine Fo_80_, plagioclase An_22_, pyroxene Wo_31_En_41_Fs_28_, and saponite), Ca sulfates (gypsum), halogen minerals (NaCl, MgCl_2_, NaBr) and (per)chlorates (NaClO_3_, NaClO_4_) were chosen as the starting dust analogues; all of them are present in martian soil [1-3]. Each starting mineral grain was well ground, sieved (grain sizes less than 150 μm) and baked at 150°C for 24-72 hours to remove adsorbed H_2_O. A fused SiO_2_ cell (with a diameter of 13 mm, inner depth of 2 mm and volume of 240 mm^3^) filled with a powder sample was used in the ESD experiments.

**1.1 The Mars chamber and ESD device**

We conducted ESD-glow discharge experiments in a Mars chamber at Shandong University, Weihai. The Mars chamber setup is shown in Figure S1. A gas control and vacuum system was regulated to adjust the gas flow and pressure. During the ESD process, the Mars chamber was evacuated to a gas pressure as low as possible (approximately 0.3 mbar) by removing the air and then filled with dry CO_2_. The flow of CO_2_ was controlled at 2 L/min by gas mass flow metres. To simulate present-day Mars, the atmospheric pressure was maintained at 3±0.1 mbar with active in-filling and exhausting of CO_2_.

The ESD-glow discharge was generated by a pair of copper electrodes (with diameters of 32 mm and a distance of 6 mm). A fused SiO_2_ cell was placed on the bottom electrode. We used an AC power supply (220 V, 50 KHz), a plasma generator (Nanjing Corona Lab Co., Ltd., model: CTP-2000K) connected to a touch voltage regulator (Zhengjiang Guangfa electric Co., Ltd., STG-500 W) that was connected to the ESD electrodes. During the experiment, the driving voltage was set to 120 V, and the electric current was ~0.22 mA. A thermocouple was used to monitor the reaction temperature in real time.

**1.2 Raman spectroscopy**

Raman spectra of the ESD products were obtained with a Reinshaw inVia Raman system with 532 nm excitation and a spectral resolution of ~1 cm^-1^, and the experiments were performed at Shandong University, Weihai. A 50x objective lens with a long working distance was used to focus the laser beam on the sample, and the beam diameter was less than 1 μm. The laser beam energy for our measurements was approximately 5 mW. Before each measurement, a standard Si wafer was used for wavelength calibration, and the positions of the Raman peak were within the range 520.7 ± 0.1 cm^-1^. For each sample, Raman spectra were collected at multiple spots in several areas. Raman spectra data were processed with WIRE 3.4 software.

**1.3 Infrared spectroscopy**

Mid-IR attenuated total reﬂectance (ATR) spectra were collected using a Bruker VERTEX 70 Fourier Transform Infrared Spectrometer (FTIR) at Shandong University, Weihai. A diamond anvil ATR accessory (PIKE Technology) was used to perform the mid-IR measurements. The sample powders were scraped off from the top layer of each ESD sample cell and then placed on the ATR sample holder. The sample was pressed against the ATR accessory to bring it into close contact with the diamond. When infrared radiation passed through the diamond and entered the sample, it was totally reflected in the frequency range that the sample did not absorb, and it was transmitted in the frequency range that the sample absorbed. We collected ATR spectra from 600 to 4000 cm^-1^ with a resolution of 4 cm^-1^. For each sample, 64 scans were averaged to generate a smooth spectrum. The background was collected before each measurement.

**1.4 CO_3_^2-^ content measurements with the double indicator method**

We applied the double indicator method [4] to determine the carbonate yield of each ESD experiment. Phenolphthalein (Aladdin; P196509-100 ml) and methyl orange (Aladdin; M196492-250 ml) were prepared as indicators. First, we rinsed the burette with a hydrochloric acid standard solution (MREDA; 0.01 mol/L) and the Erlenmeyer flask with deionized water. The burette was then filled with the hydrochloric acid standard solution and flushed to remove all air bubbles from the burette tip. Ten millilitres of deionized water and the ESD product were transferred into an Erlenmeyer flask; then, two drops of the phenolphthalein indicator were added. The hydrochloric acid standard solution was dripped into the Erlenmeyer flask until the red indicator turned colourless. Subtracting the initial volume from the final volume yielded the amount of titrant used to reach the endpoint V_1_. In this process, the reaction was Na_2_CO_3_ + HCl → NaHCO_3_ + NaCl. Two drops of methyl orange indicator were then added. The hydrochloric acid standard solution was dripped into the Erlenmeyer flask until the yellow indicator turned orange. The consumption of hydrochloric acid standard solution in this titration was V_2_. In this process, the reaction was NaHCO_3_ + HCl → NaCl + H_2_O + CO_2_. Simultaneously, the starting sample was used as a blank. The yield of carbonate was calculated according to the stoichiometry of the chemical reaction and the volume of standard hydrochloric acid consumed in the titration. The yields of Na_2_CO_3_ and the volume of hydrochloric acid consumed are listed in Table S1.

**2. Possible effects of the atmosphere and air pressure on the ESD reaction**

The real Martian atmosphere is composed of ~96% CO_2_, 1.9% N_2_, 1.9% Ar, and 0.14% O_2_ [5]. CO_2_ is the dominant component of the present-day Martian atmosphere; CO_2_ also serves as the carbon source for carbonate formation via the ESD reaction. In this study, we used nearly pure CO_2_ (99.99%) with a pressure of 3 mbar to simulate the present-day atmosphere. Additional experiments were conducted to test the possible effects of the atmosphere and air pressure on the ESD reaction.

To test the possible effects of the atmosphere, we performed ESD experiments with simulated Mars gas to compare the results with the experimental results obtained with pure CO_2_. The Mars Simulate Gas Mixture (MSGM) we used in the present study was composed of 95.94% CO_2_, 1.96% Ar, 1.94% N_2_, and 0.16% O_2_. The experimental setup and other parameters were all the same as the ESD experiments performed with pure CO_2_. In the final ESD products, carbonate and (per)chlorate were identified by mid-IR attenuated total reflectance (ATR) spectra (Figure S6). This MSGM experiment confirmed our previous results, which also strongly indicated that a pure CO_2_ atmosphere can be used to simulate the ESD reactions on Mars. Another advantage of our experiment was that using pure CO_2_ rather than other mixed MSGMs avoided interference with other gases or reaction products. This benefitted our understanding of the carbonate formation mechanism.

To test the possible effects of air pressure, we performed ESD experiments with various CO_2_ pressures. At 6 mbar, the ESD glow discharge was unstable and did not effectively induce the electrochemical reaction. For the NaCl-ESD-5 h-2 mbar products, a strong feature at ~1430 cm^-1^ and a weaker band at ~ 883 cm^-1^ (Figure S7) for Na_2_CO_3_ were identified. However, carbonate was not identified in the NaCl-ESD-5 h-1 mbar products, and carbonate was not effectively detected in the final products of the NaCl-ESD-5 h-1 mbar and NaCl-ESD-5 h-2 mbar experiments using the double indicator method (Table S1).

The gas pressure used in this study was 3 mbar instead of 6 mar (Martian atmospheric pressure). This followed our previous experiments [6,7]. The main reason for choosing the 3 mbar pressure was that the ESD CO_2_ glow discharge can only be steadily generated at pressures of 1~3 mbar in the Mars chamber. We would like to emphasize that the different CO_2_ pressures did not change the reaction path of the ESD plasma reaction. The relatively low pressure produced intense ESD glow discharge, which increased the efficiency of the ESD chemical reactions. Clearly, this aided qualitative and quantitative analyses of the final products.

Based on the above experiments and previous studies [6], we concluded that pure CO_2_ and a 3 mbar pressure can be used to simulate the ESD processes occurring on present-day Mars and understand the complex chemical reactions caused by the ESD.

**3. Estimation of CO_2_ sequestered by the EDS process**

Here, we attempted to estimate the amount of CO_2_ sequestered by the EDS process in the Amazonia era. Based on the data acquired by the Gamma Ray Spectrometer (GRS) aboard the NASA 2001 Mars Odyssey mission, the average abundance of Cl on the Mars surface is ~0.49 wt% [8]. If all Cl was accounted for by chloride salts (e.g., halite) and putting aside oxychlorides, the estimated NaCl content for the Mars surface was ~0.81 wt%.

Based on our experimental results for NaCl-ESD-5 hours, the fixed CO_2_ efficiency obtained in our experiments (per square centimetre; sample cup diameter 13 mm) was 4.567E-07 g/cm^2^·h. The calculation proceeded as follows:

((0.0085 mmol*44 g/mol)/(1000*3.14*0.65 cm*0.65 cm)*5)*(0.81/100) = 4.567E-07 g/cm^2^. This was taken as the efficiency of ESD dust formation on Mars realized by sequestering CO_2_.

Considering that the dust activities on Mars are seasonal and occur in local regions, we assumed the temporal probability (1%) and spatial probability (10%) of dust storms occurring on Mars in the Amazonian era (3.0 Ga) [9]. Based on the work of Jakosky et al. [10], a 100-m thickness for a global dust layer equates to 44 g CO_2_/cm^2^ at ~18 mbar of CO_2_. This provides approximately 1.37 g/cm^2^, which is equivalent to approximately 0.56 mbar of CO_2_. The calculation was as follows: 1.37(g/cm^2^)/44(g/cm^2^) *18 mbar=0.56 mbar.

However, there is large uncertainty with respect to the total amount of CO_2_ sequestered during Mars dust storms. Many key parameters remain poorly constrained, such as the efficiency of CO_2_ conversion to carbonate, Martian atmospheric pressure variations, the contents of chloride and (per)chlorate in Martian dust, and the potential catalytic effects of other minerals. These variations have major impacts on the amount of CO_2_ sequestered by EDS processes over the geological timescale.

**4. Figures, tables and their captions**

Figure S1: Schematic diagram of the Mars chamber used for the ESD experiments.

Figure S2: Raman spectra of the starting phases and ESD products.

Figure S3: Mid-IR spectra of the starting phases and ESD products.

Figure S4: Mid-IR spectra of the MgCl_2_ and ESD-5 h products.

Figure S5: Mid-IR spectra of different materials and the ESD-5 h products.

Figure S6: Mid-IR spectra of the NaCl-5 h-1 mbar and 2 mbar products under pure CO_2_.

Figure S7: Mid-IR spectra of the NaCl-ESD-5 h products from the Mars Simulate Gas Mixture (95.94% CO_2_, 1.96% Ar, 1.94% N_2_, and 0.16% O_2_).

Table S1: The yield of Na_2_CO_3_ and the volume of hydrochloric acid consumed.


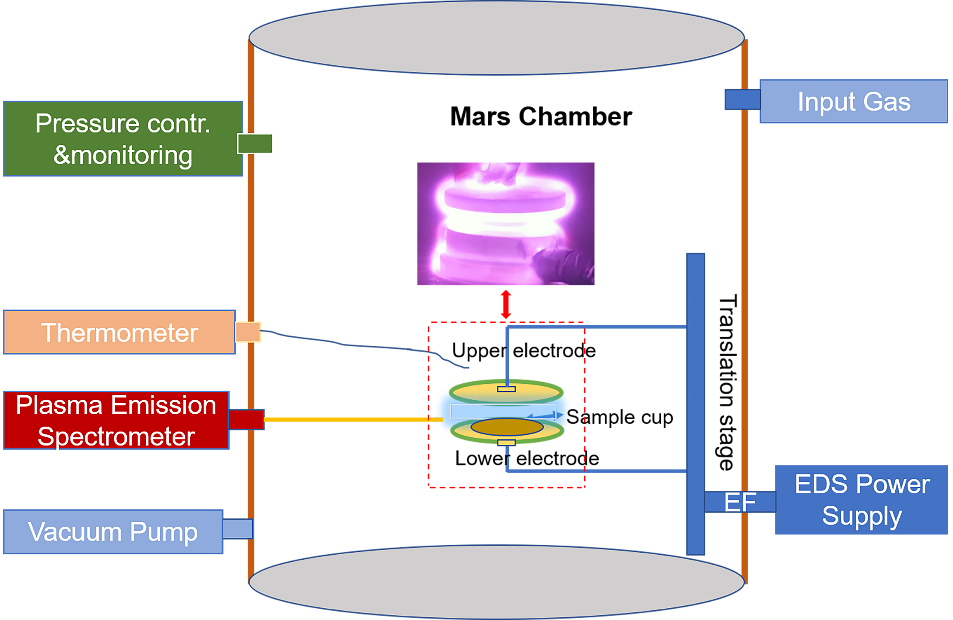


Figure S1. Schematic diagram of the Mars chamber used for the ESD experiments with an inset photo of the two copper electrodes showing a plasma generated by the ESD. A SiO_2_ glass cell filled with powdered sample rested on the lower electrode. ‘EF’ represents electronic feedthrough.


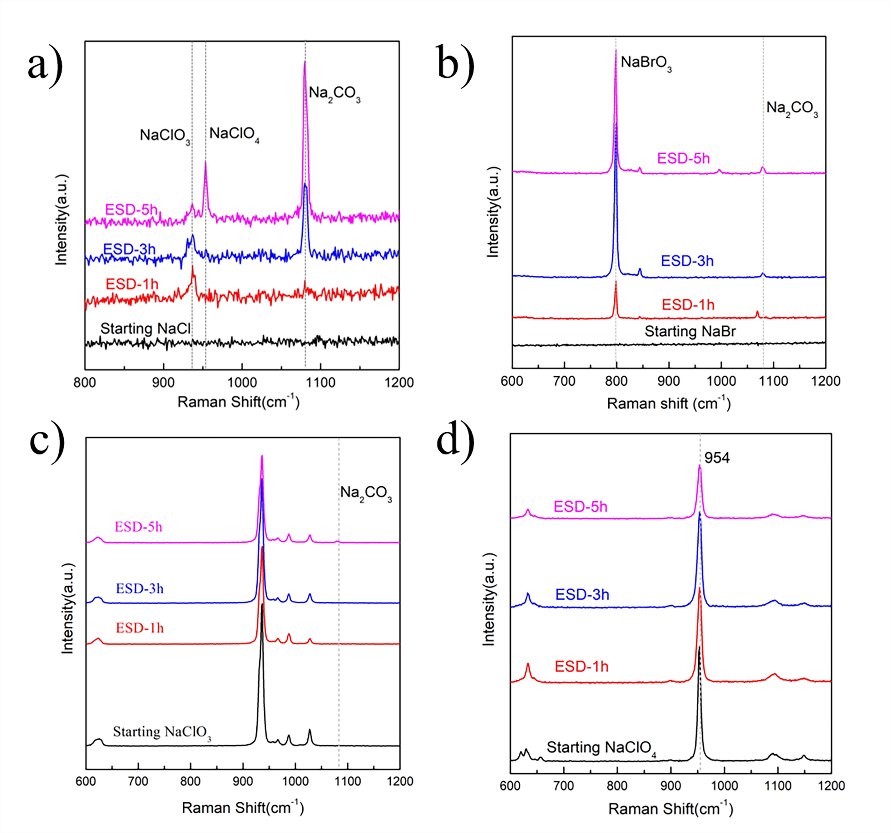


Figure S2. Raman spectra of the starting phases and ESD products. a) Starting NaCl and ESD products; b) starting NaBr and ESD products; c) starting NaClO_3_ and ESD products; d) starting NaClO_4_ and ESD products. The carbonates, oxychloride and oxy-bromine newly formed after the ESD showed sharp Raman peaks in the range 750-1200 cm^-1^.


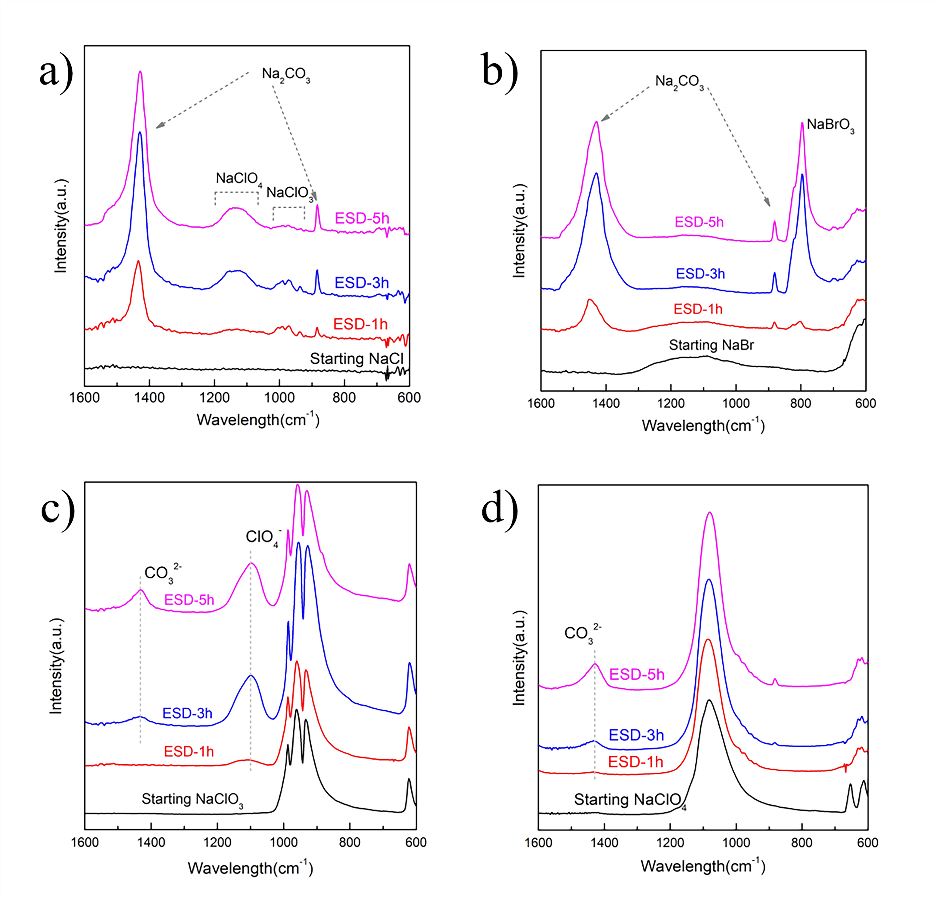


Figure S3. Mid-IR spectra of the starting phases and ESD products. a-d) ESD products with various starting dust precursors: NaCl, NaBr, NaClO_3_, and NaClO_4_, respectively. Note that carbonate and oxychloride/oxybromide resulted from different starting minerals and times during the ESD process.


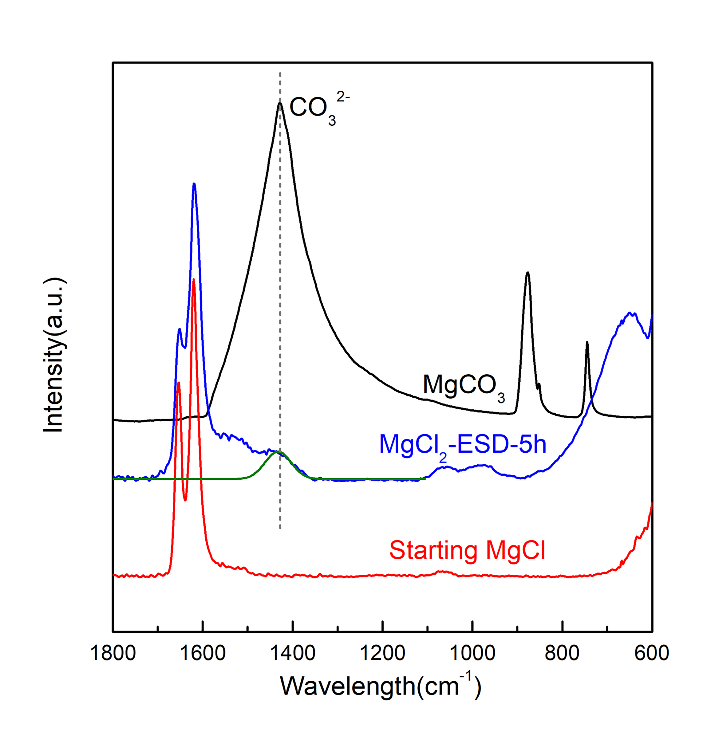


Figure S4. Mid-IR spectra of the MgCl_2_ and the ESD-5 h products. CO_3_^2-^ was detected in the experiments using MgCl_2_ as the starting material. Mid-IR spectra of MgCO_3_ (RRUFF); reference spectra are shown for comparison.


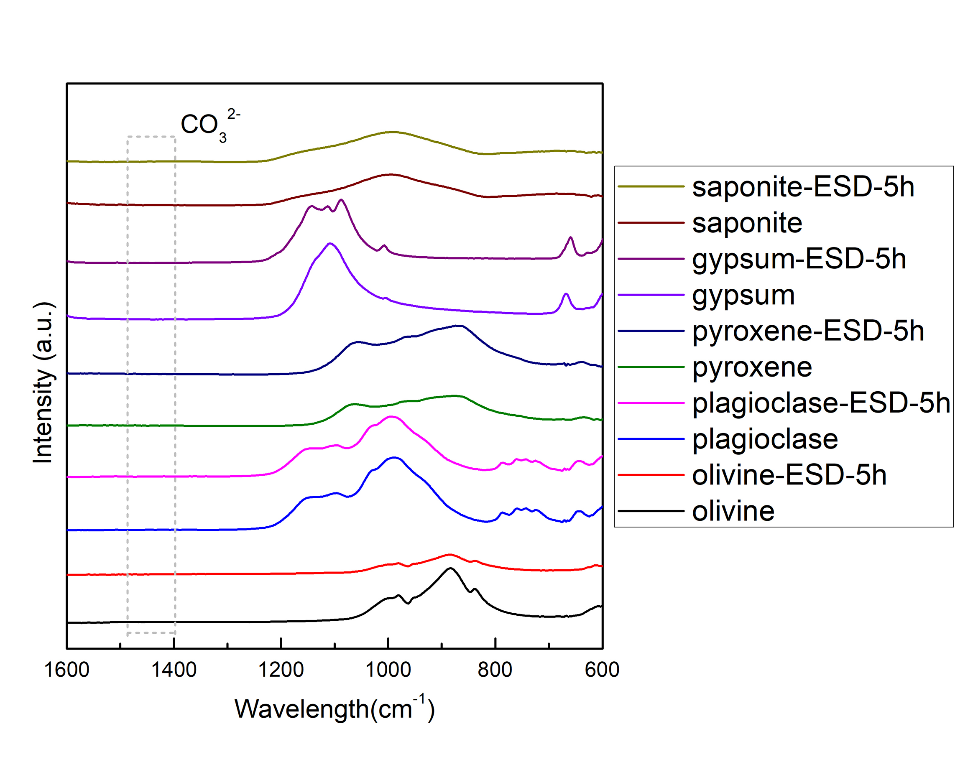


Figure S5. Mid-IR spectra of different materials and the ESD products, olivine, plagioclase, pyroxene, gypsum and saponite. In the experiments using silicates (olivine, plagioclase, pyroxene, and saponite) and gypsum as the starting materials, CO_3_^2-^ was not detected in the final products.


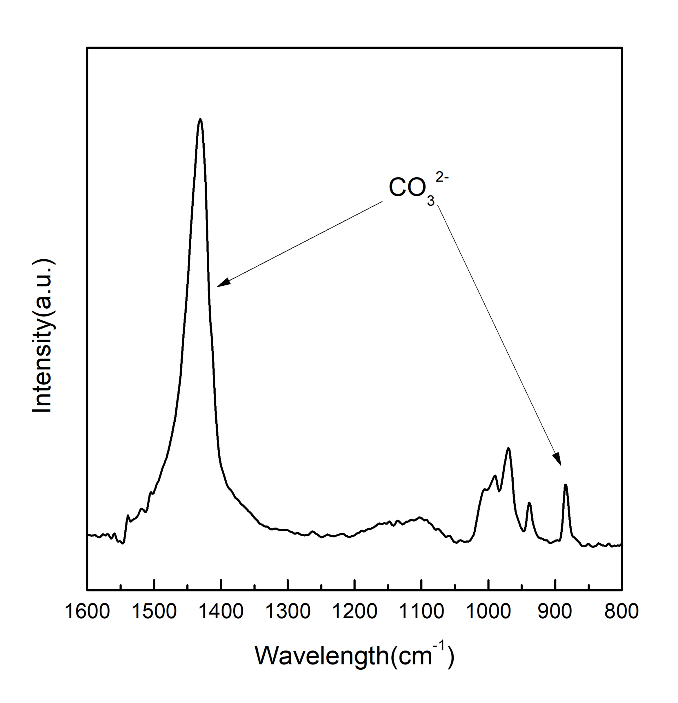


Figure S6. Mid-IR spectra of the NaCl-ESD-5 h products from the Mars Simulate Gas Mixture (95.94% CO_2_, 1.96% Ar, 1.94% N_2_, and 0.16% O_2_). The characteristic vibrational features of CO_3_^2-^ at 1430 and 883 cm^-1^ were observed.


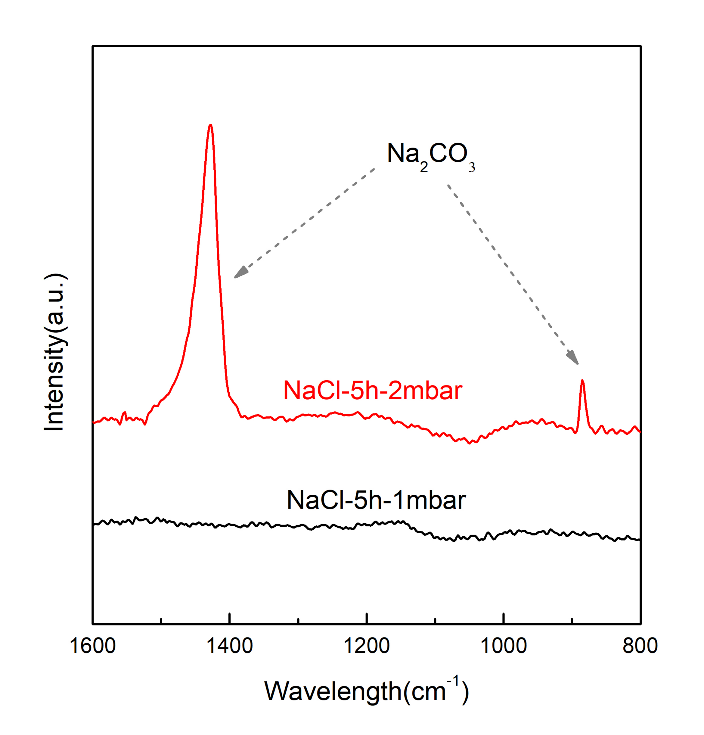


Figure S7. Mid-IR spectra of the NaCl-5 h-1 mbar and -2 mbar products formed under CO_2_. The newly formed CO_3_^2-^ was detected only in the products from the NaCl-5 h-2 mbar experiment.

Table S1. Yields of Na_2_CO_3_ and volumes of hydrochloric acid consumed.

| Sample | V_1_ (ml) ^a^ | V_2_ (ml) ^b^ | Sample weight (g) | Molar of  Na_2_CO_3_ (mmol)^c^ | Na_2_CO_3_ (wt.%) |
| --- | --- | --- | --- | --- | --- |
| NaCl | 0 | ~0.1(2 drops) | - | - | - |
| NaBr | 0 | ~0.1 (2 drops) | - | - | - |
| NaClO_3_ | 0 | ~0.1 (2 drops) | - | - | - |
| NaClO_4_ | 0 | ~0.1 (2 drops) | - | - | - |
| NaCl-ESD-5 h-1 | 0.85 | 1.10 | 0.2775±0.0001 | 0.0085 | 0.32±0.036 |
| NaCl-ESD-5 h-2 | 1.00 | 1.20 | 0.2665±0.0001 | 0.0100 | 0.40±0.037 |
| NaBr-ESD-5 h-1 | 2.35 | 2.45 | 0.3791±0.0001 | 0.0235 | 0.66±0.026 |
| NaBr-ESD-5 h-2 | 2.40 | 2.60 | 0.4136±0.0001 | 0.0240 | 0.62±0.024 |
| NaClO_3_-ESD-5 h-1 | 0.30 | 0.35 | 0.2533±0.0001 | 0.0030 | 0.13±0.039 |
| NaClO_3_-ESD-5 h-2 | 0.30 | 0.40 | 0.2463±0.0001 | 0.0030 | 0.13±0.040 |
| NaClO_4_-ESD-5 h-1 | 0.30 | 0.35 | 0.1723±0.0001 | 0.0030 | 0.18±0.058 |
| NaClO_4_-ESD-5 h-2 | 0.22 | 0.30 | 0.1928±0.0001 | 0.0022 | 0.12±0.051 |
| NaCl-ESD-5 h-1 mbar-1 | 0 | ~0.1(2 drops) | - | - | - |
| NaCl-ESD-5 h-1 mbar-2 | 0 | ~0.1(2 drops) | - |  |  |
| NaCl-ESD-5 h-2 mbar-1 | 0 | ~0.1(2 drops) | - |  |  |
| NaCl-ESD-5 h-2 mbar-2 | 0 | ~0.1 (2 drops) | - | - | - |

^a^ V_1_ is the volume of hydrochloric acid from the beginning of the titration up to the point when the phenolphthalein indicator changed colour. ^b^ V_2_ is the volume of hydrochloric acid from the point where the phenolphthalein indicator changed colour up to the point when the methyl orange indicator changed colour. ^c^ In Table S1, V_1_ is approximately equal to V_2_, and moles of Na_2_CO_3_ produced= C_(HCl)_(V_1+_V_2_)/2/1000.

**References**

1. Rampe, EB, Blake, DF, Bristow, TF, et al. Mineralogy and geochemistry of sedimentary rocks and eolian sediments in Gale crater, Mars: a review after six Earth years of exploration with Curiosity. Geochemistry. 2020; 80(2).

2. McLennan, SM, Anderson, RB, Bell, JF*, et al.* Elemental geochemistry of sedimentary rocks at Yellowknife Bay, Gale crater, Mars. *Science*. 2014; **343**(6169).

3. Rieder, R, Gellert, R, Anderson, RC*, et al.* Chemistry of rocks and soils at Meridiani Planum from the alpha particle X-ray spectrometer. *Science*. 2004; **306**(5702): 1746-9.

4. Rice, EW, Baird, RB, Eaton, AD. Standard methods for the examination of water and wastewater, 23th edition. *American Public Health Association, American Water Works Association, Water Environment Federation*. 2017.

5. Mahaffy, PR, Webster, CR, Atreya, SK, et al. Abundance and Isotopic Composition of Gases in the Martian Atmosphere from the Curiosity Rover. *Science*. 2013; 341(6143): 263-6.

6. Wu, Z, Wang, A, Farrell, WM*, et al.* Forming perchlorates on Mars through plasma chemistry during dust events. *Earth and Planetary Science Letters*. 2018; 504: 94-105.

7. Mao, WS, Fu, XH, Wu, ZC*, et al.* The color centers in halite induced by martian dust activities. *Earth and Planetary Science Letters*. 2022; 578.

8. Keller, JM, Boynton, WV, Karunatillake, S, et al. Equatorial and midlatitude distribution of chlorine measured by Mars Odyssey GRS. *J Geophys Res-Planets*. 2006; 111(E3): 18.

9. Martin, LJ, Zurek, RW. An analysis of the history of dust activity on Mars. *J Geophys Res-Planets*. 1993; 98(E2): 3221-46.

10. Jakosky, BM. The CO_2_ inventory on Mars. *Planetary and Space Science*. 2019; 175: 52-9.
